# A Review of Traditional, Time-Honoured Foods and Recipes: To Choose to Use or Not to Use

**DOI:** 10.3390/foods14193371

**Published:** 2025-09-29

**Authors:** Victor Benno Meyer-Rochow

**Affiliations:** 1 Department of Ecology and Genetics, University of Oulu, SF-90140 Oulu, Finland; meyrow@gmail.com; Tel.: +358-413-183648; 2 Agriculture Science and Technology Research Institute, Gyeongkuk National University, Andong 36729, Republic of Korea

**Keywords:** traditional diets, nutrients, food history, international dishes, ingredients, health

## Abstract

Traditions in connection with foods do not just refer to the kinds of food consumed, but also to the place and society they originated from and the ways the items to be consumed were obtained and prepared. There is a tendency to believe that what people ate in former times was more natural, nutritionally superior and generally healthier than what we include in our diets nowadays. Although this is true for some foods, it is not correct for all foods and one needs to be critical and accept that even in the past some, dangerously unhealthy and nutritionally deficient food items and diets existed and that in our modern society we avoid various foods for a variety of reasons. On the other hand, in addition to age-old methods to preserve foods and make them palatable, we developed new ways to increase the shelf life of our food products and learned to improve taste and quality of some of our foods. Some dishes and ingredients are used to highlight important occasions and commemorative events. This paper critically evaluates the nutritional and cultural significance of traditional foods and explores the factors influencing their acceptance or rejection among contemporary consumers. By re-evaluating the cultural and nutritional roles of traditional foods, this paper contributes to ongoing discussions on health, identity, and sustainability in the global food landscape. Moreover, it functions as a conceptual lens to help decide which of the traditional foods and beverages to keep, modify, or discard.

## 1. Introduction

Traditions pervade our lives from start to end. They are involved in how and where we are born, they influence how we dress, how we speak and how we learn and they affect the music we prefer listening to, the books we choose to read, the sports that we enjoy, the games we like to play, the manners we observe and, of course, the food that we eat. The question of what the essence of a traditional food is has recently been revisited by Rocillo-Aquino et al. [1]. These authors in their multidimensional study in which they contrast traditional food with typical, regional, ethnic, and local food conclude that its main characteristic is to transmit “knowledge and raw materials between generations”. This agrees with observations by Ramli et al. [2] on food heritage and food identity. The word ‘tradition’ is a weighty word as it evokes historical roots, respect given to ancient customs and habits, to authenticity and a sense of ‘belonging’— and yet, considering food and beverages and the ways they are prepared, aren’t times, fashions, tools and equipment changing and isn’t hanging on to traditions sometimes more of an encumbrance than an asset [3]? It depends, of course, on the kind of tradition, the situation and the context.

Regarding foods and recipes, it is important to know the history of a dish, its ingredients, the region it stems from, when it is consumed and how its consumption affects society, the environment, flora and fauna. Taking a closer look before deciding what and what not to accept, an examination of the way the food looks, smells, tastes and feels (see Ghosh et al. [4]) may be required. Sensory cues such as these are involved in deciding whether to accept or to reject a particular food and how well it is to be remembered. In summary, the consumer needs to be informed and that is what this essay attempts to achieve; however, not by presenting an exhaustive list of traditional foods of the world, but by using appropriately selected examples.

## 2. Foods of the Past

Traditions in connection with food not only involve the kind of food consumed, but also the place from where the food item is obtained. Traditions also include the ways the food is collected, harvested, stored or, in the case of animals, hunted, trapped, killed, preserved and made into something palatable. Traditions at all levels can weaken or be modified with time, and may even disappear altogether, while habits and preferences change and new foods as well as technologies appear. There is a tendency to assume that our ancestors with no access to processed and sugar-containing food other than perhaps honey or aphid secretions [5] lived healthier lives, feasting on ‘natural nutrition’. However, although they undoubtedly had a much lower refined sugar intake than modern people today and did not know “junk food”, it is a fallacy to believe that their food was protecting them against all kinds of diseases. As pointed out by Trueba and Dunthorn [6], a variety of tropical diseases can be traced to the palaeolithic era or even before.

It has been documented that despite their traditional food intake, our ancestors also suffered from osteoarthritis, rickets, headaches, cancer, hernias, bladder stones and a surprising amount of dental decay [7]; even diabetes was not unknown [8]. Shortages of certain vitamins were also not uncommon, e.g., vitamin D or B_1_ deficiencies and a lack of minerals, e.g., iodide salt, etc. There were of course also the not necessarily diet-related diseases such as cholera, typhoid, leprosy, smallpox rabies, malaria, tuberculosis, trachoma and afflictions associated with old age such as brittle bones, weakening eyesight and hearing loss; yet, without doubt, prehistoric folk suffered far less from obesity-related illnesses that we observe these days [9,10].

The relative lack of cardiovascular disease and type 2 diabetes in prehistoric populations, often thought to be related to the food prehistoric people ate, has recently come under scrutiny by Gurven et al. [11], who pointed out that chronic infections, by flatworms and especially nematodes obtained through unwashed or undercooked food, may have offered protection “against heart disease and diabetes through direct and indirect pathways. As part of a strategy to ensure their own survival and reproduction, helminths exert multiple cardio-protective effects on their host…” The helminths’ consumption of blood, the latter with its content of lipids and glucose, would have altered the hosts’ metabolism and modulated their immune function, which then could lead to “lower blood cholesterol, reduce obesity, increase insulin sensitivity, decrease atheroma progression, and reduce [the] likelihood of atherosclerotic plaque rupture” [11].

### 2.1. When to Speak of Foods with a Tradition and When of Traditional Foods

Although the example given above does not mean that we should now also infect ourselves with helminth worms and consume unwashed and undercooked food, it does show that we have to be careful in uncritically praising the benefits of traditional foods and food habits. While some food traditions are useful and should be preserved for a variety of reasons, not all food and preparation-related traditions are worth keeping; some are certainly of value and have stood the test of time while others should not be revived or followed. What is positive particularly holds true for food stuffs and dishes known to bolster health and fitness of an individual, but it may also involve time-honoured preparation methods and connections with what is available during different seasons or times of food shortages. After all, what suits special social or family events of a person’s life best, and what is affordable or morally acceptable at this time and age in our modern society differs from the past. Considering traditional food stuffs and ingredients in our spectrum of food can broaden our food repertoire and reduce the focus on only a small number of high-yield crop varieties and animal species [12]. Traditional foods are neither always nutritionally recommendable (the diet of the Onabasulu based largely on sago of the sago palm tree comes to mind [13]) nor always very diverse, as according to Hill and Hurtado [14], 98% of the calories in the diet of the Ache, a traditional people of Bolivia, were supplied by only 17 different food items out of hundreds of available food sources.

As Meyer-Rochow [15] has explained, not all foods that go under the label “traditional” are actually traditional and, secondly, as also pointed out above, not all traditional foods are worth preserving as they may have become socially and morally unacceptable, are detrimental to our health and well-being, may have a bad effect on the environment or have now become so expensive that their nutritional value does not compensate for the money spent on them. Most discussions skip the problem of having to define what is meant when people discuss traditional foods or foods with a tradition. The latter need not be associated with a particular people or culture or linked to a region but are traditions that arose in several places independently in connection with times of hardship, stress or food shortages. Traditions of using the bark of a variety of trees as food or a source of nutrients can be found amongst peoples around the world. Similarly, the use of various insects during famines has been a traditional habit in many parts of the world (which is not to say that certain insect species have not become, or were, not used in some cultures as a traditional food item for the insect’s exotic and appealing taste for which wealthy people were, and still are, prepared to pay a large amount of money).

#### 2.1.1. Foods with a Tradition

An excellent example for a food item with a tradition that falls short of the definition of a traditional, i.e., regionally and seasonally restricted food, or a food affiliated with a particular culture or religion, are perhaps the so-called “ship’s or sea biscuits” [16], also known as “hard tacks” [17]. This kind of dry cracker made of barley, rye or bean flour keeps for a long time and had been used as sustenance by sailors worldwide on long journeys and soldiers during wars with restricted access to fresh food stuffs. Hard tacks as a food item are not associated with the traditional food of a people, country, culture or region, but represent a widely appreciated food item with a long tradition, but certainly not a traditional food.

#### 2.1.2. Beverages with Tradition

There are also beverages that fall into this category. All of the world’s dark brown ‘Colas’ have a history that goes back to 1886 and John S. Pemberton’s original Coca Cola [18], which itself has a history that has roots in liquids containing coca leaves of *Erythroxylum coca* [19,20] and caffeine-containing kola nuts, originally from West Africa [21] that Amerindians in especially Peru, but also Argentina, Bolivia, Colombia, and Ecuador were and are consuming as a herbal tea. Therefore, to call the world’s varieties of ‘colas’ a drink with a tradition rather than a traditional drink would be appropriate (which is not to say that traditional herbal teas and other drinkable liquids, including alcoholic ones, do not exist: they do, of course). A hot drink, substituting coffee beans with roasted and ground oak acorns or barley, is the caffeine-free German *muckefuck* coffee (from the French *moka foux* = fals mocha: [22]). Traditionally consumed by people with different ethnic and cultural backgrounds in various countries during times when real coffee was unavailable or too expensive, this ‘false coffee’ has seen some increase in popularity recently because of its absence of caffeine and can also rightly be called a drink with a tradition rather than a traditional potation.

#### 2.1.3. Beyond Solid Food and Beverages

**Figure 1:**
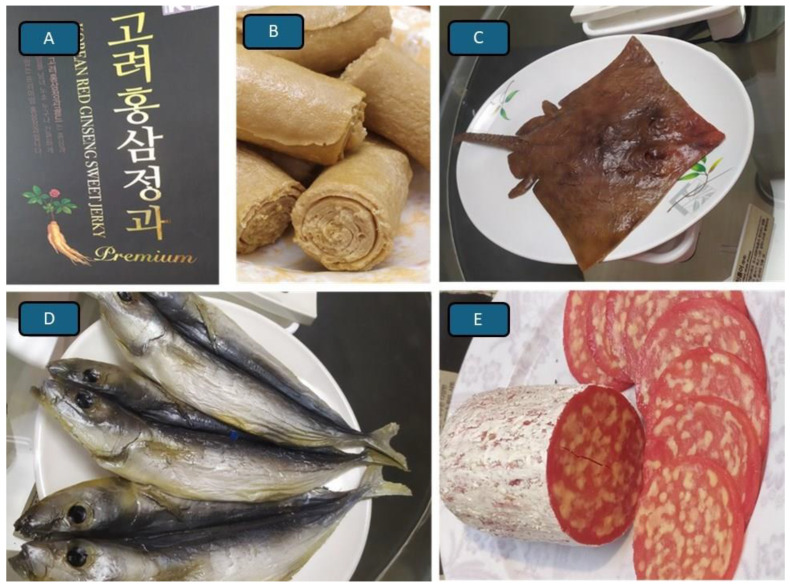
Traditional foods from around the world. (**A**). Korean *ginseg* is used as a traditional food ingredient and medicine. (**B**). Fermented Ethiopian and Eritrean flatbread known locally as *injera*. (**C**). A small, fermented ray, also known as skate (Elasmobranchii; Rajidae). (**D**). Fermented fish sold in Sweden as ‘*surströmming*’ and Japan as ‘*kusaya*’. (**E**). Hungarian salami as an example of the many kinds of ‘*wurst*’ or ‘*salami*’.

Some food items like chewing gum reveal similar patterns in how traditions may emerge and evolve. Chewing gum is certainly not consumed as a food and only accidentally swallowed, but one could nevertheless wonder whether chewing gum is a tradition, perhaps from the USA, or a widespread practice with a tradition. If so, a tradition of whom, restricted to which part of the world, with a long history? Since mastic gum of the mastic tree (*Pistacia lentiscus*) has already in the Antiquity served the inhabitants of Greece, Turkey, Syria and Lebanon as an early form of swallowable ‘chewing gum’ [23] it more likely would classify as a practice with a tradition. Traditional foods and foods with a tradition are clearly not the same but are frequently linked to medicinal uses [24]. The Korean ‘ginseng’ springs to mind (Figure 1A), an antioxidant containing root of the genus *Panax* used in various foods and drinks because of its credited with improving brain function, erectile problems, blood sugar management, and is beneficial for immunity and cancer: even a Journal of Ginseng Research exists. Food seen and used as medicine is even more apparent with the methods of food preservation [25,26], of which drying, salting, smoking, fermenting, and conserving food by heating and sealing it in air- and watertight containers are known throughout the world. Freezing or preserving food in cool places has traditionally been known only by inhabitants of cold countries (see also [27]).

### 2.2. Fermentation as a Tradition

The items that are subjected to the process of fermentation vary and the results are often traditional and highly regionally distinctive food products (Table 1). Controlled fermentation [28] is a global tradition to turn fruits, seeds and some vegetables, or honey, milk and cheeses as well as fish and meat into something with quite different, regionally distinctive tastes and textures (Figure 1B–E). As such, the procedure of fermentation involves the actions of yeasts (e.g., mostly *Saccharomyces cerevisiae*, but in some cases also *Geotrichum candidum*, *Kluveromyces marxianus* and *Pichia fermentans*), and additionally numerous species and strains of milk and lactose-associated bacteria. Starter microbes include lactic acid bacteria (e.g., *Lactococcus lactis* or *Lactobacillus* spp.) and *B. subtilis* for natto (non-LAB) and others involved in making yoghurt and sauerkraut). The process of fermentation is typically a part of producing a huge variety of foods, some that can rightfully be called traditional, e.g., those depicted in Figure 1B–E.

A detailed list of fermented foods from different regions of the world, is given in https://en.wikipedia.org/wiki/List_of_fermented_foods (accessed on 14 April 2025), an article, which is richly illustrated and distinguishes the fermentation of (a) beans (including soy products), (b) types of cheese, (c) condiments, (d) milk-based cremes, (e) grain-based foods, (f) fruits and vegetables, (g) meats and seafood, as well as (h) beverages. Some widespread and well known examples in each category would be (a) bean curd, i.e., tofu (China), miso and natto (Japan), ssamjang (Korea); (b) Limburger (Belgium), Tilsiter (Russia), Parmesan (Italy); (c) Ganjang (Korea), Iru (Nigeria), Sumbala (West Africa), Vinegar, Worcestershire sauce; (d) Crème fraîche (France), Skyr (Iceland), Viili (Finland), Yoghurt; (e) Brem (Indonesia), Dosa (India), Pitha (India), Idli (India), Injera (Eritrea) and Ethiopia); (f) Atachara (Philippines), Kimchi (Korea), Pickles, Sauerkraut (Germany); (g) Cod liver oil, Fermented fish, Igunaq (N. American and N. East Asian Arctic cultures), Kusaya (Japan), Salami (Hungary and Europe generally), Som moo (Thailand), Surströmming (Sweden).

One of the world’s most popular and widespread foods that employ the fermentation process are breads (https://www.youtube.com/watch?v=C8U23AS1irY) (accessed on 14 April 2025) of which a large number of very traditional varieties exist, e.g., to name but a few those based on rye such as black bread (popular in Nordic and East European countries) and pumpernickel (popular in Germany), ciabatta (Tuscany, Italy), sourdough bread (various European regions), cornbread (Latin America), leavened flatbreads like Naan (India), Challah (Jewish traditional bread), pumpkin and carrot containing breads. *Injera* is an Ethiopian and Eritrean fermented spongy flatbread (Figure 1B), which uses teff flour, a product that comes from the grains of *Eragrostis tef*, a kind of cereal. As with the *injera* rolls, the shapes of the breads are often also traditional and may be long or round as in the respective cases of the French baguette or boule and may be sprinkled with a variety of spices or seeds as with sesame bread rolls, poppyseed or cummin seed rolls, etc. Traditions, as with the new kinds of bread rolls, can evolve and this has also been shown in a new study by Danmek et al. [29] who explored the potential of adult worker honey bees (*Apis mellifera* L.) as a sustainable protein source for a new fermented sauce using *Aspergillus oryzae*, diverging from traditional plant-based condiments.

Some of the most famous beverages, which include highly traditional drinks as well as drinkables with a long tradition, all depend on the fermentation process and beers of a wide variety, *calpis* (Japan), *chicha* (Latin America), *kefir* and *kumis* (Central Asia), *lassi* (India), *mead* (China, Greece, Europe), wines of a wide range based on different grapes and the areas they are cultivated, and other crops including vegetables such as rice, apples, plums, cherries, currants, elderberries, pomegranate, carrots, and potatoes, etc., come to mind. The end products may contain ethanol (beers, wines, liquors), are either alcohol free when fresh (*lassi*, *calpis*) or may contain moderate (*mead*, *cider*) or small amounts of alcohol (*kefir*, *kumis*, *chicha*).

### 2.3. Traditional Foods and Dishes

As Vanhonacker et al. [30] suggested in 2010 and Rocillo-Aquino et al. [1] also emphasized recently, what we categorize as a “traditional food” or dish is based mainly on the perspective of the European consumer. The whole issue is clouded further by conceptual distinctions such as “established”, “culturally embedded”, “ethnic” and “regionally invented” or “local” and “typical”. As the categories are overlapping, to give a clear, generally acceptable definition for each term is therefore simply not possible.

#### 2.3.1. Traditionally American?

Three examples that Meyer-Rochow [15] chose to illustrate this dilemma with were the American “hamburger”, the famous “American apple pie”, and the traditional meal served on the day of “Thanksgiving”. One could ask if there was anything more traditionally American than these three foods. But are they in fact traditional American foods? Approximately 150 years ago, the American ‘hamburger’, a kind of ‘fast and easy food’ consisting of placing a beef meat patty between the two halves of a bisected bread roll, made it from the port city of Hamburg, where it was known as a “*Rundstueck warm*” to the New World. In the USA, a certain Charlie Nagreen of Wisconsin [31] is credited with having “invented” the American version of it in 1885 (although it has also been claimed that Fletcher Davis came up with his “hamburger” in the 1880s in Texas: [32]). Can we now truthfully decide whether the ‘hamburger’ is a traditional American food or does the traditional ‘hamburger’ belong to the city of Hamburg? What if someone claimed it came to Hamburg from the port city of Bremen? I dare not answer that, because my ancestors hailed from Bremen. Obviously, cultural exchanges and how they affected knowledge, distribution and acceptance of unfamiliar food around the world make it really difficult to define what counts as a “traditional food”.

Perhaps the apple pie and roast turkey for Thanksgiving provide us with better answers of what is, at least, traditionally American. But problems arise immediately, because according to Eschner [33], the apple tree (*Malus domestica*) is native to Eurasia and could have come to America only with the settlers from Europe. In addition, pies such as the apple pie existed in several European countries long before the apple pie became popular in the USA. And the “Thanksgiving turkey”, a bird that even sparked an annual tradition in the American “White House”: a time-honoured tradition? What is certainly known is that the turkey is a tasty bird, native to America and that it is usually served with mashed potatoes and cornbread and green beans or pumpkin for Thanksgiving. There are dozens of often slightly regionally different recipes, some containing pumpkin and apple-walnut stuffing and the cranberry sauce and others do not. However, would European settlers in America have regarded turkey, potato, corn bread, pumpkin and beans as part of their European-based traditional diets? They would not, because all of these “Thanksgiving Meal” associated food items are of American and not European origin [34]. Is the present-day “Thanksgiving Dinner” therefore a traditional American food, given that traditionally Amerindians (and not even the early European immigrants) would not likely have consumed it? This most famous of traditionally American food would therefore qualify only as a food that became a tradition amongst immigrant Americans and settlers, but not as an indigenous traditional food.

#### 2.3.2. Traditional, but with Foreign Ingredients

**Figure 2:**
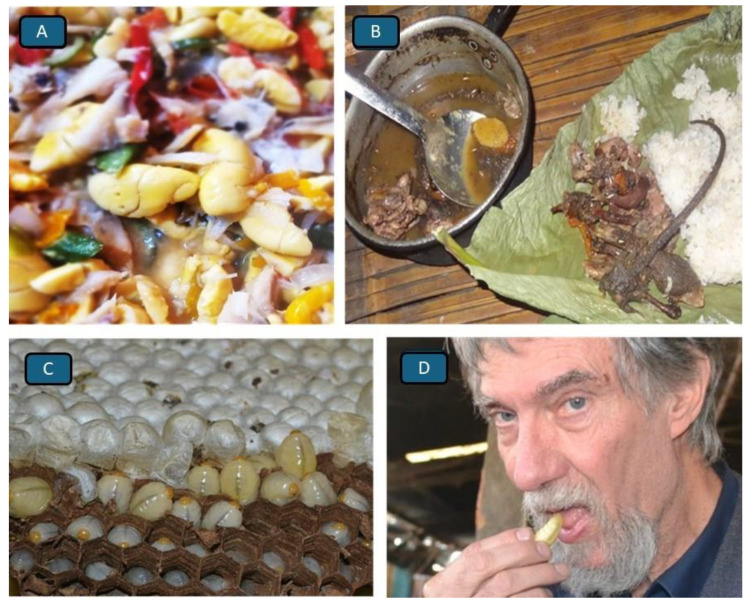
Traditional foods from around the world. (**A**) “*Ackee and Saltfish*”, the traditional dish of Jamaica. The yellow pieces are the fruit flesh of the fruit of the ackee tree (*Blighia sapida*). (**B**) The “*bule-bulakoying*” dish of the Adi in North-East India contains the meat, innards, heart, feet and tails of field rats served as a highly appreciated meal. (**C**) Larvae of the giant Asian hornet *Vespa mandarinia* are some of the most expensive (and tastiest) edible insects in Asia. (**D**) *Vespa mandarinia* larvae may be consumed raw, fried, boiled or soaked in ethanol.

A similar objection could be raised in connection with so-called traditional foods that are now on offer in countries in which the ingredients for these foods did not originally exist and had not been used in the past. One could come up with a whole list of fruits, nuts and vegetables, of spices and even fish and meats, which made it into so-called traditional dishes, but those containing potatoes, sweetcorn, pumpkin, tomatoes, various beans, etc., and then served with pork, beef, mutton, etc., strictly speaking, could hardly have been traditional as the essential ingredients had either earlier been unknown or become available only after being imported from abroad. The definition of what is traditional is what we constantly have to grapple with if we do not wish to be misunderstood and it is often more appropriate and accurate to call a dish “established”, or “time-honoured and popular” or simply just “nowadays very common”. Whether we can assign a food to a particular ethnic group or call it a ‘tribal food’ or link it to a particular region is equally difficult and the Jamaican tradition of consuming “Ackee and saltfish, served with rice” (Figure 2A) shows that beautifully well.

Jamaicans are, to an overwhelming degree, of West African ancestry, but sadly tribal affiliations to a country or specific region of origin are largely lost. It is therefore simply not possible to link a particular dish to a specific African tribe or region [35]. The ackee is an orange-coloured fruit of the *Blighia sapida* tree, which stems from West Africa, and which was taken to the Caribbean where it became established. The saltfish, an essential component of Jamaica’s traditional dish “Ackee and Saltfish”, is in most cases air-dried marine fish of the cod family Gadidae, known as stockfish, imported from Norway. The rice is not native to the Caribbean and is of South and Southeast Asian origin. Although none of the basic ingredients are of a Jamaican origin, the dish is widely regarded as Jamaica’s traditional food and loved by none other than the world’s fastest man: Usain Bolt.

One could argue that even though the ancestors of present-day Jamaicans would not have known “Ackee and saltfish served with rice”, it was the special way Jamaicans put the three ingredients together, which is then sufficient justification for the food to be called a traditional dish. So-called traditional foods that are based on (or contain) rice like, for example, the *joulupuurot* (various kinds of Christmas rice puddings in Finland: [36] are also known from countries that never cultivated or could grow rice. However, since local people learned to use and prepare rice in ways that often differed from those in the rice-growing countries, the situation is similar to that of Jamaica’s Ackee and Saltfish and one could then justify the term traditional food.

### 2.4. Ethnic Foods

#### 2.4.1. Ethnic or Traditional?

Distinguishing between ethnic and traditional foods is not always possible, but generally “ethnic food” is more closely related to a specific ethnic group, irrespective as to whether the ethnic group still resides in the region of its origin, while traditional foods can but need not be associated with a particular ethnicity. Based on this definition, the Scottish “*haggis*” and the Adi People’s “*bule-bulakoying*” are unarguably ethnic foods, but the haggis, at least, could equally well be called a traditional food as it is nowadays also enjoyed by non-Scots [15]. The main ingredients of a “haggis”, which is accepted to be of Scottish origin although similar varieties are known from other regions, are a sheep’s heart, liver and lungs. Often regarded as offal and easily perishable, these organs are minced with oatmeal, onion, suet, spices and salt and then cooked inside a sheep’s stomach. The mixture is served with swedes and potatoes (the latter of course originally taken from South America by Columbus to Europe and then widely grown and appreciated in Scotland). The Scottish haggis is popular with Scotts in places as far away from Scotland as New Zealand and it creates a common bond between people of Scottish ancestry.

Another truly traditional food is the “*bule-bulakoying*” dish (Figure 2B) of the Adi people of North-East India [37]. The Adi have been catching wild rats with ingeniously constructed traps for times immemorial and know how to turn the rodent into a delicious and nutritious meal for humans. As a food item, wild rats are popular in many parts of the Earth, but amongst the Adi, thousands of rats are consumed during the unying-aran hunting festival on March 7th each year. The bule-bulakoying dish contains the boiled rat’s tail, legs, heart, liver, testes or foetuses and other inner organs and is served as a stew with leafy vegetables and rice. It is the most highly appreciated rat meat dish known to the Adi and definitely deserves to be called traditional, just like the Croatian “Stone Soup” and ‘*bikla*’, a drink prepared with goat milk and red wine (for various recipes see: [38] as well as *urumiit* consisting of the droppings of the snow ptarmigan (*Lagopus muta*) and reported to be collected locally in the high Arctic’s winter season by some Inuit of northern Canada and Greenland [39]. The Chinese bird-nest soup [40] is frequently referred to as an extremely expensive traditional dish, mainly served during Lunar New Year festivities, which involves nests made from solidified saliva of swiftlets collected in a variety of South Asian countries.

#### 2.4.2. Traditional, but from Where?

**Figure 3:**
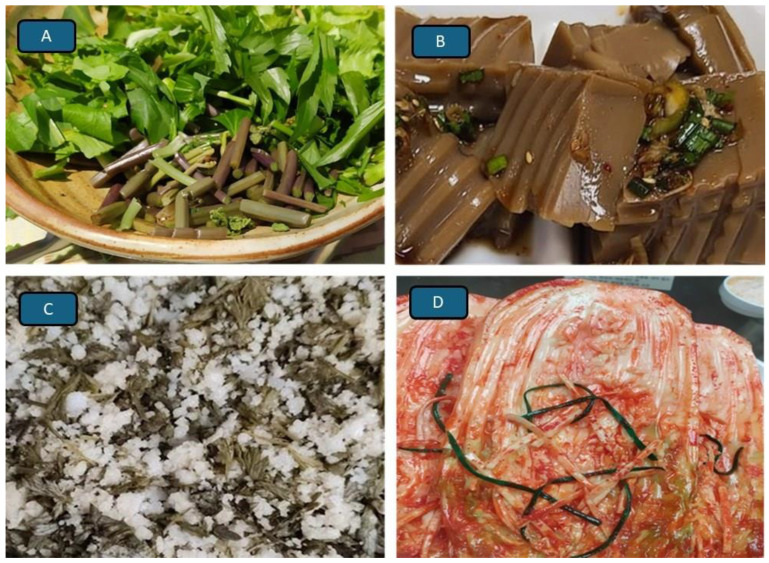
Traditional foods around the world. (**A**) Traditional Korean salad of bracken stems (front) and mugwort leaves. (**B**) No longer just a poor person’s food in Korea: jelly made from oak acorns. (**C**) Traditional East Asian rice cake with ‘*ssuk*’ leaves (*Artemisia princeps*). (**D**) Fermented cabbage ‘*kimchi*’, now increasingly common in the West.

**Figure 4:**
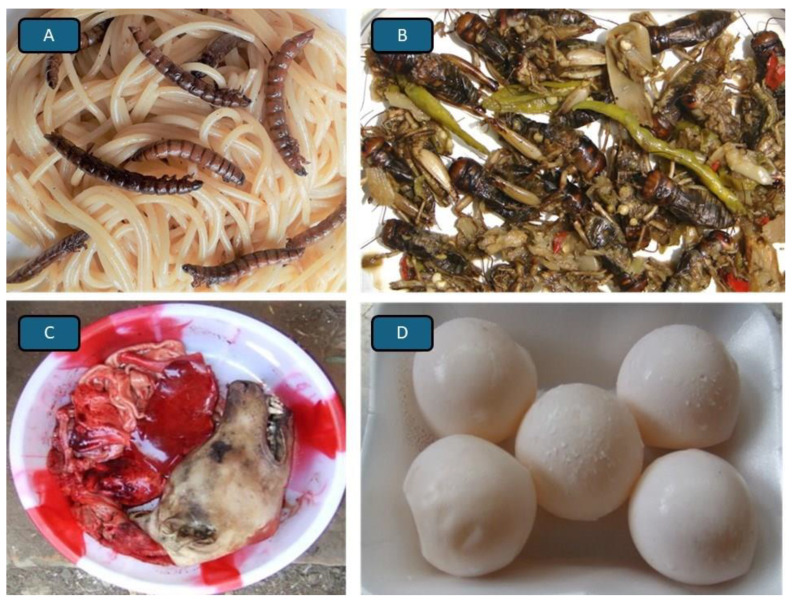
Traditional foods from around the world. (**A**) A fusion of traditions: spaghetti with *Tenebrio molitor* beetle larvae. (**B**) Indian pickles with crickets, but other insects may also be used. (**C**) Dog meat offered for sale (from January 2027, slaughtering dogs and selling their meat will be illegal in the Republic of Korea—as it is already in most countries). (**D**) Turtle eggs (and one ping pong ball in the centre for comparison) are now still legally available only in a few countries or regions.

The consumption of wasp larvae by many inhabitants of South and East Asian countries (Figure 2C,D) or bracken, acorn jelly, mugwort rice cake, and kimchi by Koreans (Figure 3A–D) is often termed a traditional practice, a diet steeped in history. However, as should have already become clear from the examples presented earlier, to define what *is* traditional and which dish, meal, or food *deserves* to be called traditional is far from easy. The consumption of insects by traditionally living people (do such people actually still exist, is the big question) is often referred to as a traditional practice. However, there are hundreds if not thousands of different edible insects, occurring in different countries, climates and habitats, subjected to different preparation techniques and being consumed at different times and for different reasons. To call edible insects a “traditional food” is therefore imprecise and misleading, unless additional information is provided, e.g., as with Italian spaghetti containing mealworms (Figure 4A) or Indian pickles containing crickets (Figure 4B).

## 3. A Variety of Diets

### 3.1. Organic Food

In spite of the difficulties to define what is meant by “traditional food”, using the search words “Organic Foods, Traditional Foods, Ethnic Foods, Traditional Diets; and Traditional Nutrition” will yield a vast number of hits. Even though food is always organic, unless one includes salt and other minerals as “food”, what is nowadays often inferred to by using the adjective ‘organic’, is that the food in question was produced without synthetic fertilizers, pesticides and genetic modification. And, furthermore, that livestock are not supposed to have been given hormones or antibiotics. Food and beverages subscribing to these stipulations are also frequently referred to as ecological or biological foods and seen by many as a healthy alternative to foods not fitting the definition of an ‘organic food’. But are they always a healthier alternative? Let us examine.

#### 3.1.1. The Vegetarian Diet

Various kinds of diet have been promoted in the past [41], of which the vegetarian diet is perhaps the most popular diet with millions of followers worldwide and especially high numbers in India, where the belief that you are what you eat is particularly strong. Although there appears to be some truth in that, it needs to be pointed out that both Mahatma Gandhi as well as Adolf Hitler (the latter apparently only in his later years) were vegetarians. One distinguishes strict fruit and vegetable-based vegetarianism from lacto-ovo vegetarianism that in addition to fruit and vegetables permits the consumption of eggs and milk products. Our prehistoric ancestors just like our primate relatives, the great apes (gorilla, orangutan, and chimpanzee), were mostly frugivorous and leaf-eating vegetarians for which their metabolism, their dentition, intestine and enzymes as well as their ability to distinguish colours (carnivores are largely colour blind) were optimally adapted, but who, as at least chimps have shown, would also not refuse to eat shellfish, insects, eggs and occasionally partake in the consumption of meat.

#### 3.1.2. The Vegan Diet and the Carnivore Diet

The vegan diet is a more extreme form of special food item avoidance, as it shuns all animal-derived products including milk and honey, but accepts plant-based substitutes and minerals. The carnivore diet is the opposite to the vegan diet as it champions a food, based entirely on animal products, devoid of plant fibres. Its value was recently assessed by Goedeke et al. [42], who concluded that it met various nutrient reference values, but fell short in vitamin B_1_, thiamin, and vitamin C as well as folate, the minerals magnesium and calcium, iron, iodine and, in some cases, potassium. The carnivore diet is somewhat similar to that of the Inuit (based on the traditional Eskimo diet: [43]), which consisted largely of fish and meat, but also contained the plant-derived, vegetarian stomach content of reindeer and musk oxen and berries in the summer.

#### 3.1.3. The Palaeo Diet, Carbohydrate-Rich Diet, and Mediterranean Diet

The so-called Palaeo diet is an omnivorous diet with few carbohydrates, no sugar and no dairy products. Tooth decay was virtually absent in people with this diet. Cardiovascular and other age-associated diseases was also not a major problem of the Japanese Okinawa islanders [44,45], the Melanesian Trobriand Islanders [46] and the Bolivian Tsimane, who all reach an old age with robust cardiometabolic health and whose diet is dominated by high energy, complex carbohydrate and protein intakes in the ratio of 3:1 and low fat intake for the Tsimane [47,48] and even 10:1 for the Okinawans [49]. In the traditional Mediterranean diet [50], minimally processed plant-based foods such as fruits, vegetable salads, pasta, nuts, milk products and small amounts of fish or meat dominate. Other diets such as the Atkins diet, and Jenny Craig diet as well as diets for purely medical reasons, e.g., gluten free, diabetic, and liquid, exist but are less common.

### 3.2. Good and Bad Diets

#### 3.2.1. Plants and Mushrooms to Be Avoided Because of Their Toxicity or Rarity

Although traditional diets such as those of the Okinawa, Kiriwina and Tsimane people, which are based highly on unrefined carbohydrate-rich food stuffs such as rice (Poaceae), yams (Dioscoreaceae) and sweet potatoes (Convolvulaceae), and appear to be very beneficial, not all traditional diets can be recommended or sanctioned (Table 2). Those that can be deadly without following a strict protocol in preparing and turning them into nutritious and, in some cases, delicious foods are those that contain the red berries of the yew tree (*Taxus baccata*). The tree is known throughout Europe and North Africa and has been introduced to North America. The best time to harvest the fleshy, red berries is late summer in Ireland, Northern and Central Europe. Since the seed of the fruit is highly poisonous and can kill a person when ingested, only the sweet and delicious fruit flesh is usable and has to be separated from the seed. Yew berry jam (of course without the deadly seeds) has a fantastic flavour enhanced by adding a bit of lemon juice. The berries are rich in vitamin C, several vitamin B kinds, and minerals such as potassium and magnesium. Traditional desserts, cakes, pastries and candies in Arab countries and India may be tasty but can be unhealthily saturated with refined sugar and can turn into health hazards if eaten too frequently.

Other examples of traditional but poisonous if inadequately prepared traditional foods, are in New Zealand the *karaka* fruit as it can cause paralysis. However, baked and water-soaked karaka berries made into cakes or made into a wine, are not at all dangerous. The seeds of cycads, an ancient group of plants, are also highly poisonous but for the traditional food of the Anami in Japan and Australian Aborigines, to name but few, the fruit is rendered edible by removing the toxin through brief leaching in water, prolonged leaching in water and subsequent ageing [51]; in some cases, roasting or drying of seeds aged for several months is used. The earlier mentioned ackee fruit of the *ackee* tree in Africa and Jamaica can kill the consumer if it is not boiled a few times in water with the water then discarded. Bracken is consumed traditionally by, to name but a few, New Zealand Māori and Koreans (Figure 3A), who eat the rhizomes or stems, respectively. Without such treatments raw bracken may be carcinogenic.

Outright poisonous fruit and plant species are widely known and recognized and not used in traditional dishes, but that rhubarb leaves can make the consumer sick and fenugreek seeds are unhealthy [52] but used in traditional foods is less well known while mugwort (*Artemisia princeps*) is widely used in Chinese, Korean and Japanese cuisine to prepare dumplings, soups, kimchi, and rice cakes with it (Figure 3A,C). It is also used to add flavour to desserts, beverages, ice creams, teas and ssuk latte, but in Europe the mugwort species *Artemisia vulgaris* is known to contain a substance called thujone, which can be toxic in large amounts and cause hallucinations and seizures, (especially in *A. absinthium* as explained by Eadie [53]). Amongst plants used in traditional foods that can be harmful for another reason are those with overly hot tastes like chilli peppers known as the Carolina Reaper (*Capsicum chinense*) or the Mexican habanero, ancho or guajillo chillies. They are frequently a component of the traditional Mexican and Texan dishes such as ‘Chilli con Carne”, and although they are rich in antioxidants [54], they need to be mentioned and should not be given to little children.

Considering traditional foods that have to be treated with caution because of their possibly deadly effects on the consumer, those containing certain species of mushrooms need to be mentioned. One of them (and there are numerous species), *Gyromitra esculenta*, is potentially fatal if eaten raw, but it can be rendered edible when parboiled and is esteemed in the traditional creamy False Morel Soup of Finland for its rich flavour. Ink heads, also known as inky caps (*Coprinus* spp., *Coprinopsis atramentaria*) are components of numerous mushroom-containing dishes. They can be delicious and healthy [55] when prepared immediately after being collected, but must not be consumed with alcoholic beverages, as then its consumption can cause coprine poisoning with vomiting, palpitations and diarrhoea.

A category of traditional foods that may be avoided for moral reasons, because they may become extinct if not protected, can affect both plants and animals. This is best shown by the disappearance of *Silphium*, a fennel relative, which was so appreciated in Greco–Roman cooking and as a medicinal herb that it became extinct [56].

#### 3.2.2. Animals and Their Parts to Be Avoided Because of Their Toxicity or Ethical Dilemmas

One of the most poisonous animals used in traditional foods are puffer or globe fish [57], served in Japan as *fugu*, in Korea as *bok* and in China as *hetun*. The fish contains tetrodotoxin, a substance that blocks sodium channels and has been of invaluable use in elucidating physiological properties of the nervous system, as it stops signal transmission, paralyzes the muscles while the victim, which remains conscious, eventually dies from asphyxiation [58,59].

For moral reasons, species nowadays to be avoided as food items, include the wild, not cultured, common European sturgeon (*Acipenser sturio* Linnaeaus). This species has disappeared from all European rivers except the Loire and the Gironde-Garonne-Dordogne system in France. Incidentally, the related Yangtze sturgeon was declared extinct in the wild in 2022. Pet species, such as dogs (Figure 4C) as well as highly vulnerable animal species that are still being used as components of traditional foods include marine turtles (Figure 4D), several species of shark of which only their fins for shark fin soup are collected (a disturbing and cruel practice that sees the live shark returned to the sea without its fins), the giant Chinese salamander (*Andrias davidianus*), various African bush meat species [60] including the pangolin, sea conch and, in southern European countries such as Italy, France, Malta, etc., small songbirds such as the ortolan bunting *Emberiza hortulana*. Although protected in most of Europe, it is still possible to find the birds served illegally in the traditional cuisine [61]. Recently, the practice of cutting up live octopus or consuming live octopus known in Korea as ‘*sannakji*’ has come under scrutiny as being unacceptably cruel and in New Zealand, Norway and Switzerland, it is now illegal to put conscious rock lobsters into boiling water [62].

Whale meat has been a traditional food item for the inhabitants of various countries for centuries if not millennia and their numbers worldwide have slowly been recovering so that it cannot be argued that whales are on the verge of extinction. However, some people avoid whale as well as dog meat, a traditional food in several countries that have recently restricted or prohibited its sale but not its consumption (Figure 4C). For some people, dishes containing horse meat or horse meat sausages are unacceptable, even though they are traditional in many countries including France, Germany and many other European regions.

Foie gras, a delicacy in France, that involves force-feeding geese to fatten up their livers, raw fish Sashimi cut from living fish and known in Japan as ‘*ikiizukuri*’, the boiling of live lobsters, etc., are traditional practices that many consumers find unacceptable, although frequently the same consumers may have no qualms to suck up a live oyster, to feast on veal or lamb, in other words meats of mammals killed only hours or a few days after being born, and piglets roasted on a spit. Raw fish, especially freshwater species, and dishes with edible snails, such as the small Jamaican river snail (*Neritina punctulata*), known locally as *bussu*, can contain parasites such as helminths and are often rejected by Westerners. The latter also frequently react with disgust when a traditional drink is offered to them, which is known as *kavakava* [63] in parts of Polynesia or yaqona in Fiji and which in some regions used to be prepared by women spitting chewed kava root paste into a bowl where it then fermented to yield the alcohol. There can, of course, be other reasons to avoid or refuse traditional foods, namely when they had involved forced or child labour, or excessive cruelty to animals.

## 4. Traditional Foods and Factors Involved

### 4.1. Climate, Geography and Habitat

Traditional food involving marine fish and clams are not very likely to be found amongst dwellers of mountainous terrain and people whose home is the tropical jungle will in all likelihood have not made plants and animal species into traditional dishes if they had not been occurring locally. Obviously, the geographic location where a specific fruit, vegetable, spice, fish or meat hailed from and became a traditional food is an important issue and now, with worldwide trade links, an even greater one. Locally restricted, traditional foods of a region are under threat and new foods, supported by smart marketing campaigns, are constantly making inroads. However, traditional foods and drinks can also expand their range, and excellent examples are the Japanese “*sushi*”, the South American quinoa (*Chenopodium quinoa*), the Korean “kimchi” and the South African rooibos tea (*Aspalathus linearis*), as well as the East Asian spirits *sake*, *soju* and *shochu* moving westward [64] and wine expanding its popularity in the opposite direction. Much earlier it was the pizza that as a traditional food of a certain region made it globally as a widely accepted and successful dish with an Italian origin.

However, traditional foods of a region are often surprisingly resistant to change, even if a people have moved to a new geographic location. Karelians of Finnish ancestry now residing in the Tver region near Moscow still search for and consume mushrooms like their forebears in Finland have done [65]. If a familiar species was no longer available, people in their new environment did not abandon their traditional food or recipe, but switched to locally available material [66] and European settlers from the Netherlands, Germany and the U.K. in Namibia, who could not find their familiar herring for “*kippers*” or German style “*matjes*”, simply began to use the common pilchard, a herring relative. Further, there is vodka, which is known to have existed before the arrival of the potato in Europe from Peru around the mid-1500s: when wheat was in short supply and expensive, first Polish and then Russian peasants began to use potatoes to produce the spirit [67].

That traditional foods also strongly depend on the climate is when we look at the Indian *dhosa*, whose North Indian variety varies dramatically from the South Indian version. Climate and weather-related differences in traditional food stuffs are known from all regions of the world and can involve vegetables and fruits, bird eggs, fish and game animals (cf., Mexico: [68]; Zaire/Congo: [69]; Japan: [70]; Southeast Asia: [71]. Most obvious in connection with traditional foods is the connection between food insect species and environmental temperature. Making sure that the correct species for insect-containing dishes are available at the market before the cooking can start, has been reported from Cameroon by Muafor et al. [72] and for Nagaland by Mozhui et al. [73] and Kiewhuo et al. [74]. Australian Aborigines, who in search of the appreciated bogong moth (*Agrotisinfusa*), used to carry out annual collecting trips in the summer months into the mountains of New South Wales (Australia) to gather the aestivating (=summer-resting) moths, are a famous example of how traditional foods could affect a tribe’s behaviour [75]. The Aborigines would then have de-winged the moths and pounded them into pulp, which was then roasted and consumed as a nutritious meal.

As the example with the Australian Aborigines and their moth-colleting migrations in the past demonstrates, to know the history and length of time that a food has been used traditionally is important, too. Foods can become popular for a while and even be termed ‘traditional’ like the use of “spam meat” in the USA and then fade away. The same holds true for whale meat after the second world war in Japan or “loach nabe”, a traditional dish known in Japan as *dojou* and Korea as *mikkuragi* and *chueotang* [76] wih a slender little fish that was, and still is, common in rice paddies (*Misgurnus anguillicaudatus*), but now has lost much of its appeal. Edible insects, with an ancient tradition as a food item for humans represent the opposite. Once widely consumed on Earth [77,78], insects as a traditional food item became completely marginalized in Europe, but after it was proposed by Meyer-Rochow [79] that insects could help easing the problem of global food shortages and he suggested that the WHO and the FAO support the idea, insect-containing foods are having something of a comeback.

### 4.2. Religious Associations and Specific Consumers

Since edible insects are already mentioned in the Bible [80] and the Quran also sanctions the consumption of certain locusts as ‘halal’, it is obvious that beliefs and religions can affect what can and what cannot be eaten. Very precise guidelines of what can go into a traditional dish are given to Jews and Muslims and in almost all societies clerics, leaders, chiefs, landowners do not always consume the food of the “common people”. In societies with distinct social strata and in places where men have greater power than women, traditions often require men and women to enjoy different foods. This inequality between people, as unacceptable as it may seem, has the positive effect that pressure on certain food stuffs is diverted to more than just one highly appreciated food.

Borowiecki [81] writes that a traditional banquet of the Romanov Tsars in Russia might include “caviar, pheasant tongues, boiled bear paws or moose lips in sour cream” and other items unimaginable as regular food items for the peasants of the country. It should be mentioned, however, that it was not just the rich and famous who had access to special foods, because certain foods were also traditional in connection with some professions or activities as, for example, the *Labskaus* [82], a dish containing salted meat or fish, potatoes and onion, often also with beetroot, and served to sailors in northern Germany. Models, artists, truckers, bankers, soldiers, nuns, sportsmen (e.g., sumo wrestlers), etc., can also have their own traditional foods and special dishes.

#### 4.2.1. Special Dishes for Festivals

Traditional foods and dishes have special roles to play as re-enforcers in virtually all religions and especially on certain days or festivals. Chapter 4 by Meyer-Rochow [15] explains in detail the many traditions that involve foods for Muslims during the month of Ramadan, dishes consumed by Jews on Sabbath days, Passover, and Rosh Hashana, food acceptable to Christians during the time of lent, or at Easter and Christmas, and then there is the major Hindu festival of Diwali with its lights and ‘Lakshmi Puja’ and loads of traditional sweets [83] like *barfi*, *ladoos*, *rasgulla*, *halva*, *jaggery*, etc. In Nordic countries, special dishes and drinks are provided for midsummer night (in Finland known as Juhannuspäivä) and in Japan the New Year food is called *osechiryori*, which consists of black beans, omelette, mashed sweet potatoes, dried fish and a fish-based sausage known as *kamaboko* [84]. Traditionally served is a noodle soup known as *toshikoshi soba* and soft rice cakes known as *mocha* as well as *yakizakana* (grilled fish). In China and Korea, the lunar New Year is celebrated with a host of traditional dishes, to name but a few fish, dumplings, spring rolls, *tangyuan* sweet rice balls, good fortune fruit plate, longevity noodles and *niangao* rice cake for China [85]; rice cake soup, kimchi dumplings, jeon-pancake, stuffed mushrooms, fried zucchini and *bulgogi* beef stew for Korea [86]. Moreover, mainly the Chinese celebrate the mid-autumn festival all over the world with traditional, round moon cakes and family reunions [87].

#### 4.2.2. Foods Served on Family Celebrations

Religious festivals are one occasion when people hold family reunions, but there are other auspicious events that bring people together and create an opportunity, if not the necessity, for preparing, serving and consuming traditional dishes. Weddings are celebrated the world over with foods that frequently have a symbolic meaning [88], are special to mark the event and are usually popular with those attending the event. Wedding menus vary widely from country to country and region to region and a few examples from places around the world are described in Meyer-Rochow [15]. Something sweet is often incorporated and some food that symbolizes male and femaleness as well as prosperity is usually part of the meal as well. Food restrictions rather than a host of traditional dishes are what expectant mothers are confronted with, but after the birth of a child the event is celebrated in many countries with traditional foods for the mother and close relatives. However, far greater numbers of traditional foods as well as food restrictions accompany the passing-away of a person. Some mourners may not feel like wanting to consume anything [89], while others are given comfort foods [90]. It is interesting that Halva (which is enjoyed in India at any time) is associated in Turkey and nearby countries with a time of mourning, while something round like lentils or eggs or bread rolls symbolize the cycle of life for Jewish mourners. In Kyrgyzstan, Muslim mourners prepare a traditional bread in sizzling oil, a practice that is meant to involve not just the mourners but the spirit of the dead as well, because the smell and smoke from the frying oil rises up to the sky [91].

Of course, many other family occasions exist that warrant the serving of a traditional snack or dish. We might think of a welcoming party for someone who had been away for a while or a celebration of winning a prize, a medal, a competition or of having been promoted. For adults, if the religion permits, the tradition often includes consuming food with alcohol, but for the children in Europe the first day at school is a day with candies and other not necessarily wholesome but sweet things, preparing them for the years ahead of them.

### 4.3. Health Concerns and Traditional Foods

Nowadays, more and more people select food with a view to its nutritional value and its impact on the environment. People have become more health conscious. However, as pointed out earlier in this paper and also in [15], people of different regions, cultures and backgrounds have different metabolisms. In many countries of East Asia, adults exhibit lactose intolerance and, furthermore, cannot metabolize ethanol because they lack the enzyme aldehyde-dehydrogenase, a problem much less prevalent amongst Europeans. For some people, a carbohydrate dominated diet is wholesome as with the Tsimane or the Okinawans [48,92] (see also earlier); for others, the palaeolithic diet works. The one thing that is obvious, however, is that junk food, based on processed ingredients, containing high amounts of sugar and saturated fatty acids and being rich in calories but low on essential amino acids, minerals and vitamins cannot be a nutritionally balanced, healthy food. It is for this reason that the trend to include traditional foods based on wholemeal, black or brown rye bread, unpolished rice, etc., in one’s diet can only be supported. In fact, the traditional porridge, beans and lentils are experiencing a comeback and in Korea acorn jelly known locally as ‘*dotorimuk*’, and often earlier regarded as a food for the poor, now has become a luxury that consumers are prepared to pay a lot for (Figure 3B).

Another concern of many relates to the environment and the fact that more and more land is required to raise cattle [93]. This led to a vastly increased production of non-dairy milks, based on soybean, almonds, oats, rice, etc., and to the number of followers of the vegan or vegetarian diets. To make doubly sure that some foods they buy are uncontaminated, some consumers deliberately search for lettuce or a cabbage which shows signs that a caterpillar has nibbled on their leaves or select some carrots that still have dirt sticking to it. Sadly, many supermarkets, especially in East Asian supermarkets, present all their vegetables and fruits sealed in plastic wrapping, which makes a critical examination impossible. But it shows us that the consumer enjoys handling and examining the food before she or he decides to buy it, and several examples are given in Meyer-Rochow [15].

Visual inspection of a food item, however, is but one of the ways by which a consumer selects what is acceptable to her or him. Ghosh et al. [4] find that the smell of a food, its taste and the way it feels are all involved when it comes to decide whether a food is delicious or not (Indians with traditional eating habits argue that food can only really appreciated when in addition to seeing, smelling, and tasting it, one can also feel it using ‘touch’). Obviously, other criteria such as religious dietary laws, traditions, the financial situation, age and health condition of the consumer, as well as the origin of the food item in question, play a role.

### 4.4. Traditional (Yet, with an Accidental Origin)

I wish to end this essay on traditional foods with some remarks on how culinary mistakes could lead to unexpected results and new ‘traditional’ dishes. To term a dish ‘new and traditional’ is, of course, a paradox, for how can, by definition, a tradition be something new. But it may be justified to some dishes and drinks. However, in some cases, it may be justified for dishes as well as drinks (alcoholic as well as non-alcoholic ones). When the Austrian Emperor Franz-Joseph I ordered a special dessert, but the cook made a mess of it and, then shredding it into pieces in local dialect calling it a “schmarrn”, was unwilling to serve it to the Emperor, the latter nevertheless demanded to taste it. To the great surprise of everyone, the Emperor loved it and issued an order that henceforth the dessert be prepared in exactly the same way. Thus, this dish (a kind of fluffy pancake now often containing raisins or small pieces of plums, became the traditional Kaiserschmarrn, which is still popular in Austria, Slovenia and Bavaria.

Although many more examples exist of how accidents led to new discoveries, including new recipes and preparation methods, even black tea as a product of fermentation could be mentioned, or how humans discovered how to preserve food items by observing how animals stored their food [15], I want to emphasize that it is investigating the history of foods and recipes, which makes the study of food so satisfying and enjoyable.

## 5. Afterthought

This paper has exposed the tension between traditional and modern diets, the role of religious and ethical considerations, and the impact of globalization on endangered or marginal traditional foods. We have also seen that traditional food preparations involving fermentation and preservation by smoking, drying, freezing, burying, pickling, etc., vary regionally and cooking methods, too, are not uniform. Grilling, frying, roasting, steaming, boiling, etc., can all lead to very different end results, which is one of the problems when trying to define what “a traditional food” entails and which of the traditional practices are to be recommended. Kristbergsson and Oliveira [94] in their book on “Traditional foods: history, perception, processing” avoided providing a strict definition and instead presented instructions on how to prepare foods that others might call “ethnic foods” or “foods characteristic of a certain region”.

Although there is a move worldwide to safeguard knowledge related to the history and cultural significance of foods ingredients and dishes even in countries that have often been overlooked regarding food culture [95], traditional foods find it hard to compete with new foods [3,12]. The impact of globalization on endangered or marginal traditional foods is blatantly obvious. A food item mentioned earlier, which was popular worldwide [76,77] with even Romans and Greek of the Antiquity relishing, for example, timber grubs [96], were in fact edible insects. However, when traditional food or food ingredients are modified or adopted to blend with foods from other regions, they can experience a “renaissance”; spaghetti with mealworms on offer at some European restaurants (Figure 4A) or Indian ‘pickles’ with crickets as an exotic delicacy (Figure 4B) and even fermented fish in Assam [97] come to mind.

In Europe, edible insects have experienced a certain amount of interest in recent years, driven no doubt by environmental concerns as well as by neophilia (interest in something new [98]). A similar situation applies to edible jellyfish [99,100], served traditionally in Japan as *chukka kurage* and Korea as *haepari-naengchae*. Species such as the cannonball jellyfish (*Stomolophus meleagris*) [101] are beginning to become recognized by European and North American gourmets as an acceptable kind of novel and tasty seafood. In the future, knowledge and appreciation of some traditional foods, as Niu et al. [102] pointed out for African diets, might even be relevant to tackle problems such as “food security obesity and non-communicable diseases”. However, the question will always be, as this review has emphasized, to choose which of the many diets available are to be used.

Although there will undoubtedly be always some people who “go for unfamiliar traditional dishes” out of a sense of curiosity (= neophilia), there will be others that choose the unusual food to enhance their status and prestige, and to demonstrate to onlookers that they can obtain, accept, and even enjoy (as well as afford) the “exotic cuisine”. However, this review does not deal with such motivation for grandstanding or bragging, but it does emphasize that getting to know foods and dishes that people of other cultures appreciate, is definitely something worthwhile and can be rewarding in many different ways [103]. 

## 6. Conclusions

It is important to identify the origins of certain dishes, the ingredients that they contain, the ways they are prepared and their histories. It is equally important to know their nutritional value and whether the food and its procurement are in harmony with environmental concerns such as land use, pesticide and fertilizer uses and moral or ethical principles. It is through such studies that we can then form an informed opinion as to which food traditions ought to be kept, which are falsely claimed to be traditional, and which had better be forgotten. To help us in this regard, the European Union has introduced a classification for foods and wines (https://agriculture.ec.europa.eu/farming/geographical-indications-and-quality-schemes/geographical-indications-and-quality-schemes-explained_en#traditional-speciality-guaranteed) accessed on 24 January 2025: “Protected Designation of Origin” (PDO), “Protected Geographical Indication” (PGI) and “Traditional Specialties Guaranteed” (TSG).

Some foods may also be included in the UNESCO “List of Intangible Cultural Heritage in Need of Urgent Safeguarding”, so that their unique characteristics can be promoted and will not be lost. Kimjang, the process of making kimchi as a fundamental part of traditional Korean cuisine of both South and North Korea by using salted and fermented cabbage and radish, is on the UNESCO’s list twice. Some other foods and beverages (according to https://guide.michelin.com/en/article/features/foods-on-unesco-intangible-cultural-heritage-list (accessed on 10 April 2025) have been added) now included are Malawian “Nsima”, Neapolitan Pizza, a flatbread of the Caucasus and Western Asia known as “Lavash”, North African “couscous” and Belgian Beer (although the list of the intangible cultural heritage of humanity is not actually of dishes, but cultural practices passed down from generation to generation). The list can be expected to grow longer in the future as more and more foods and recipes are in danger of becoming marginalized and forgotten. In a rapidly changing world, the preservation, adaptation, or rejection of food traditions must be guided by informed reflection—on history, sustainability, ethics, and the human need for cultural continuity. Relevant laws may be needed and, to reinforce them, regular inspections should occur, and fines for breaking the law may be necessary. This review has shown that not all traditional foods are and were healthy as they sometimes lacked essential minerals or vitamins or contained toxins. However, if it can be shown that certain specific traditional foods are not only healthier than many modern foods, and that, moreover, they do not lead to unwanted weight increased and obesity, then a lot will already have been achieved to recognize that the study of traditional foods is still an important issue and should be taken seriously.

## Figures and Tables

**Table 1:** Some examples of traditionally fermented foods and beverages from different countries or regions of the world.

Food Category	Korea	Japan	France (South Europe)	Germany (Northern Europe)	Hungary (Eastern Europe)	India	Africa	Worldwide
Vegetable & fruit based	kimchi	natto		Sauerkraut	savanyusag	pickles	kunde, kale	See also: webpage
Milk products			Cheese, yoghurt	Cheese, quark	Russia: kvess, kefir	dahi	amasi	See also: webpage
Bean & rice based	tofu	tofu, natto			turo	dosa, idli	dawadawa	See also: webpage
Grain based, e.g., breads	Var. sauces	Sauces, e.g., miso	Baguette, sourdough	Var. kinds of bread			Ethiopia: injera	See also: webpage
Meats			Saucisson sec, salami	Mettwurst, var. sausages	Salami			See also: webpage
Fish, etc.	Jeotgal	kusaya		Sweden: surströmming		Bodo/Assam: napham		See also: webpage
Beverages	Makgeolli, soju	Beer, sake	Wine, beer, cider	Beer, wine, cider	Palinka, beer Russia/Poland: vodka	lassi	W. Africa: burukutu	See also: webpage

Further information available from webpage: https://en.wikipedia.org/wiki/List_of_fermented_foods (accessed on 5 March 2025).

**Table 2:** Some examples of traditional food items whose consumption can pose ethical or health problems.

Traditional Foods to Be Avoided if Unsure of Origin and Preparation	Possibly Dangerous or Even Lethal	Possible Cause of Pain, Allergy or Injury	Can Contain Parasites Such as Helminth Worms	Ethical Dilemma, e.g., Animal Cruelty or Rare Species
Mushrooms such as *Gyromitra esculenta & Coprinus* spp.	X	X		
Cycad seeds	X			
Karaka fruit (*Corynocarpus laevigatus*)	X			
Ackee fruit (*Blighia supida*)	X			
Yaw berries (*Taxus bacata*)	X			
Chilli peppers (*Capsicum* spp.)		X		
Fish & marine species:				
Puffer fish (*Takifugu* spp.),	X			
All raw fish			X	X
Shark fins	X		X	X
Wild sturgeon			X	X
Turtles				X
Sea urchins		X		
Various bushmeat & whale				X
Giant salamander (*Andrias* sp.)				X
Ortolan bunting songbird				X
Goose liver (Foie gras)				X

## Data Availability

No new data were created or analyzed in this study.

## References

[1] Rocillo-Aquino Z., Cervantes-Escoto F., Leos-Rodrigue J.A., Cruz-Delgado D., Espinoza-Ortega A. (2021). What is a traditional food? Conceptual evolution from four dimensions. J. Ethn. Foods.

[2] Ramli A.M., Zahari M.S.M., Halim N.A., Aris M.H. (2016). The knowledge of food heritage identity in Klang Valley, Malaysia. Procedia Soc. Behav. Sci..

[3] Patel J., Tamuli A., Teli A.B. (2025). Barriers in traditional food consumption: A cross sectional study in a city of Assam state. Int. J. Home Sci..

[4] Ghosh S., Jung C., Meyer-Rochow V.B., Halloran A., Flore R., Vantomme P., Ross N. (2018). What governs selection and acceptance of edible insect species?. Edible Insects in Sustainable Food Systems.

[5] Van Neerbos F.A., de Boer J.G., Salis L., Tollenaar W., Kos M., Vet L.E., Harvey J.A. (2020). Honeydew composition and its effect on life-history parameters of hyperparasitoids. Ecol. Entomol..

[6] Trueba G., Dunthorn M. (2012). Many neglected tropical diseases may have originated in the paleolithic or before: New insights from genetics. PLoS Negl. Trop. Dis..

[7] Humphrey L.T., De Groote I., Morales J., Barton M., Collcutt S., Ramsey C.B., Bouzouggar A. (2014). Earliest evidence for caries and exploitation of starchy plant foods in Pleistocene hunter-gatherers from Morocco. Proc. Natl. Acad. Sci. USA.

[8] Lakhtakia R. (2013). The history of diabetes mellitus. Sultan Qaboos Univ. Med. J..

[9] GBD 2021 Adult BMI Collaborators (2025). Global, regional, and national prevalence of adult overweight and obesity, 1990–2021, with forecasts to 2050: A forecasting study for the Global Burden of Disease Study 2021. Lancet.

[10] Boutari C., DeMarsilis A., Mantzoros C.S. (2023). Obesity and diabetes. Diabetes Res. Clin. Pract..

[11] Gurven M.D., Trumble B.C., Stieglitz J., Blackwell A.D., Michalik D.E., Finch C.E., Kaplan H.S. (2016). Cardiovascular disease and type 2 diabetes in evolutionary perspective: A critical role for helminths?. Evol. Med. Public Health.

[12] Ghosh S., Meyer-Rochow V.B., Jung C. (2023). Embracing tradition: The vital role of traditional foods in achieving nutrition security. Foods.

[13] Toyoda Y., Todo R., Toyohara H. (2005). Sago as food in the Sepik Area, Papua New Guinea. Sago Palm.

[14] Hill K., Hurtado A.M. (1989). Hunter-gatherers of the New World. Am. Sci..

[15] Meyer-Rochow V.B., Ghosh S., Panda A.K., Jung C., Jung C., Bisht S.S. (2023). Chapter 2: Traditional foods and foods with a tradition: It’s not the same. Emerging Solutions in Sustainable Food and Nutrition Security.

[16] Moisan E. (1989). Master of the Sweet Trade.

[17] Biggers S. (2022). How to Make Hardtack the Modern and Traditional Way. https://www.primalsurvivor.net/how-to-make-hardtack/.

[18] King M.M., John S. (1987). Pemberton: Originator of Coca-Cola. Pharm. Hist..

[19] Biondich A.S., Joslin J.D. (2016). Coca: The history and medical significance of an ancient Andean tradition. Emerg. Med. Int..

[20] Valdez L.M., Taboada J., Patton M., Manion J. (2016). Coca leaves in the context of the Central Andean Wari State. Trading Spaces: The Archaeology of Interaction, Migration and Exchange; Proceedings of the 46th Annual Chacmool Archaeology Conference.

[21] Starin D. (2013). Kola nut: So much more than just a nut. J. R. Soc. Med..

[22] Kirmse J. (2021). Muckefuck—Was Steckt in Dem Amüsant Klingenden Kaffeeersatz?. https://www.kaffeeroesterei-kirmse.de/muckefuck.

[23] Kristbergsson K., Ötles S. (2016). Functional Properties of Traditional Foods.

[24] Meyer-Rochow V.B. (2017). Therapeutic arthropods and other largely terrestrial, folk-medicinally important invertebrates: A comparative survey and review. J. Ethnobiol. Ethnomed..

[25] Hardy T. (2021). Reviving Ancient Ingredients, Methods for Food Preservation. https://www.preparedfoods.com/articles/126265-reviving-ancient-ingredients-methods-for-food-preservation.

[26] Sharma N. (2025). Food Preservation. https://flexbooks.ck12.org/cbook/cbse-science-class-8/section/2.11/primary/lesson/food-preservation/.

[27] (2025). Food Preservation. https://en.wikipedia.org/wiki/Food_preservation.

[28] Alagbe E.E., Okoye G.C., Amoo T.E., Adekeye B.T., Taiwo O.S., Adeyemi A.O., Daniel E.O. (2022). Spontaneous and controlled fermentation to improve nutritional value of ikpapa beans, *Phaseolus vulgaris*. Cogent Eng..

[29] Danmek K., Ghosh S., Jung C., Wu M.-C., Maniwara P., Hongsibsong S., Klaithin K., Chuttong B. (2025). Fermentation and physicochemical properties of sauce made from adult worker honey bees (*Apis mellifera*) using *Aspergillus oryzae*. Int. J. Food Sci. Technol..

[30] Vanhonacker F., Almli V.L., Hersleth M., Verbeke W. (2010). Profiling European traditional food consumers. Br. Food J..

[31] Cohler R. (2019). How Charlie Nagreen Changed the World. https://edibledoor.ediblecommunities.com/food-thought/how-charlie-nagreen-changed-world.

[32] Cartwright G. (2009). The World’s First Hamburger. https://www.texasmonthly.com/food/the-worlds-first-hamburger/.

[33] Eschner K. (2017). Apple Pie Is Not All That American. https://www.smithsonianmag.com/smart-news/why-apple-pie-linked-america-180963157/.

[34] Crosby A.W. (2003). The Columbian Exchange.

[35] Sainsbury B. (2021). Ackee and Saltfish: Jamaica’s Breakfast of Champions. https://www.bbc.com/travel/article/20210315-ackee-and-saltfish-jamaicas-breakfast-of-champions.

[36] Mansikkamäki S. (2017). Vesikikka Toimii—Tällä Niksillä Vältät Joulupuuron Pohjaan Palamisen. https://kotiliesi.fi/ruoka/ideoita-ruoanlaittoon/vesikikka-toimii-talla-niksilla-valtat-joulupuuron-pohjaan-palamisen/.

[37] Meyer-Rochow V.B., Megu K., Chakravorty J. (2015). Rats: If you can’t beat them eat them! (Tricks of the trade observed among the Adi and other North-East Indian tribals). J. Ethnobiol. Ethnomed..

[38] Brljevic A. (2025). Eleven Weird Croatian Dishes for the Intrepid Foodies. https://www.frankaboutcroatia.com/weird-croatian-dishes/.

[39] Millman L. (2017). This Shit Is a Delicacy. https://www.vice.com/en/article/qknnnx/this-shit-is-a-delicacy.

[40] Marcone M.F. (2005). Characterization of the edible bird’s nest the “Caviar of the East”. Food Res. Int..

[41] Yannakoulia M., Scarmeas N. (2024). Diets. N. Engl. J. Med..

[42] Goedeke S., Murphy T., Rush A., Zinn C. (2025). Assessing the nutrient composition of a carnivore diet: A case study model. Nutrients.

[43] Draper H.H. (1977). The Aboriginal Eskimo diet in modern perspective. Am. Anthropol..

[44] Willcox D.C., Scapagnini G., Willcox B.J. (2014). Healthy aging diets other than the Mediterranean: A focus on the Okinawan diet. Mech. Ageing Dev..

[45] Willcox B.C., Willcox B.J., Todoriki H., Suzuki M. (2009). The Okinawan diet: Health implications of a low-calorie, nutrient-dense, antioxidant-rich dietary pattern low in glycemic load. J. Am. Coll. Nutr..

[46] Lindeberg S., Nilsson-Ehle P., Terént A., Vessby B., Scherstén B. (1994). Cardiovascular risk factors in a Melanesian population apparently free from stroke and ischaemic heart disease: The Kitava study. J. Intern. Med..

[47] Kraft T.S., Stieglitz J., Trumble B.C., Martin M., Kaplan H., Gurven M. (2018). Nutrition transition in two lowland Bolivian subsistence populations. Am. J. Clin. Nutr..

[48] Kass M. What’s on the Menu for People in the Amazon? Not the Paleo Diet. Arizona State University News 2018. https://news.asu.edu/20181206-what%E2%80%99s-menu-people-amazon-not-paleo-diet.

[49] LeCouteur D.G., Solon-Biet S., Wahl D., Cogger V.C., Willcox B.J., Willcox D.C., Raubenheimer D., Simpson S.J. (2016). New Horizons: Dietary protein, ageing and the Okinawan ratio. Age Ageing.

[50] Willett W.C. (1995). Mediterranean diet pyramid: A cultural model for healthy eating. Am. J. Clin. Nutr..

[51] Beck W. (1992). Aboriginal preparation of Cycas seeds in Australia. Econ. Bot..

[52] Ouzir M., El Bairi K., Amzazi S. (2016). Toxicological properties of fenugreek (*Trigonella foenum* graecum). Food Chem. Toxicol..

[53] Eadie M.J. (2009). Absinthe, epileptic seizures and Valentin Magnan. J. R. Coll. Physicians Edinb..

[54] Valencia-Cordova M.G., Suárez-Jacobo A., del Socorro Cruz-Cansino N., Ramírez-Moreno E., Zafra-Rojas Q.Y., Alberto-Ariza-Ortega J., Alanís-García E. (2021). Capsaicin, dihydrocapsaicin content and antioxidants properties of habanero pepper (Capsicum chinense Jacq.) oleoresin during storage. Emir. J. Food Agric..

[55] Nowakowski P., Naliwajko S.K., Markiewicz-Żukowska R., Borawska M.H., Socha K. (2020). The two faces of Coprinus comatus—Functional properties and potential hazards. Phytother. Res..

[56] Panda T., Sahu S.K., Apollo M., Mohanty R.B. (2024). The journey of cyrenaic medicinal plant Silphium: Areview. Res. J. Pharmacogn. Phytochem..

[57] Currie B.J. (2003). Marine Antivenoms. J. Toxicol., Clin. Toxicol..

[58] Cheng K.K., Ling Y.L., Wang J.C. (1968). The failure of respiration in death by tetrodotoxin poisoning. Q. J. Exp. Physiol. Cogn. Med. Sci..

[59] Lago J., Rodriguez L.P., Blanco L., Vieites J.M., Cabado A.G. (2015). Tetrodotoxin, an extremely potent marine neurotoxin: Distribution, toxicity, origin and therapeutical uses. Mar. Drugs.

[60] Lee T.-M., Sigouin A., Pinedo-Vasquez M., Nasi R. (2020). The Harvest of Tropical Wildlife for Bushmeat and Traditional Medicine. Annu. Rev. Environ. Resour..

[61] Lee J. (2022). Ortolan: The Controversial French Delicacy You’ll Likely Never Try. https://www.tastingtable.com/1078603/ortolan-the-controversial-french-delicacy-youll-probably-never-try/.

[62] Baker H. (2021). Octopuses, Squids and Lobsters Could Become ‘Sentient Beings’ in the UK. https://www.livescience.com/cephalopods-and-crustaceans-recognised-as-sentient-in-uk.

[63] Baker J.D. (2011). Tradition and toxicity: Evidential cultures in the kava safety debate. Soc. Stud. Sci..

[64] Vinepair S. (2018). The Differences Between Soju, Shochu and Sake Explained. https://vinepair.com/articles/soju-shochu-sake-difference/.

[65] Härkönen M. (1998). Uses of mushrooms by Finns and Karelians. Int. J. Circumpolar Health.

[66] Ternikar F., Thompson P.B., Kaplan D.M. (2014). Ethnicity, Ethnic Identity, and Food. Encyclopedia of Food and Agricultural Ethics.

[67] Begg D. (1998). The Vodka Companion: A Connoisseur’s Guide.

[68] Acuña A.M., Caso L., Aliphat M.M., Vergara C.H. (2011). Edible insects as part of the traditional food system of the Popoloca town of Los Reyes Metzontla, Mexico. J. Ethnobiol..

[69] Pagezy H. (1975). Les Interrelations Homme Faune de la Forêt du Zaire. L’Homme et l’Animal, Premier Colloque d’Ethnozoologie.

[70] Nonaka K., Durst P.B., Johnson D.V., Leslie R.L., Shono K. (2010). Cultural and Commercial Roles of Edible Wasps in Japan. Forest Insects as Food: Humans Bite Back, Proceedings of a Workshop on Asia-Pacific Resources and Their Potential for Development.

[71] Yhoung-Aree J., Viwatpanich K., Paoletti M.G. (2005). Edible Insects in the Laos PDR, Myanmar, Thailand, and Vietnam. Ecological Implications of Minilivestock.

[72] Muafor F.J., Levang P., Angwafo T.E., Le Gall P. (2012). Making a living with forest insects: Beetles as an income source in Southwest Cameroon. Int. For. Rev..

[73] Mozhui L., Kakati L.N., Kiewhuo P., Changkija S. (2020). Traditional knowledge of the utilisation of edible insects in Nagaland, North-East India. Foods.

[74] Kiewhuo P., Mozhui L., Kakati L.N., Lirikum L., Meyer-Rochow V.B. (2022). Traditional rearing techniques of the edible Asian giant hornet (*Vespa mandarinia* Smith) and its socio-economic perspective in Nagaland, India. J. Insects Food Feed.

[75] Cherry R.H. (1991). Use of insects by Australian Aborigines. Am. Entomol..

[76] Dokkaebi (2021). Korean Cuisine #5 Chueotang (Loach in Hot Bean Paste Soup). https://kodokkaebi.tistory.com/18.

[77] Bergier E. (1941). Peuples Entomophages et Insects Comestibles: Étude sur les Moeurs de l’Homme et de l’Insecte.

[78] Bodenheimer F.S. (1951). Insects as Human Food.

[79] Meyer-Rochow V.B. (1975). Can insects help to ease the problem of world food shortage?. Search.

[80] The Bible Leviticus 11:12. https://www.bible.com/.

[81] Borowiecki A. (2012). Opulence Beyond Measure. https://www.stalberttoday.ca/entertainment-news/opulence-beyond-measure-1280002.

[82] Dieck T. (2013). Pottkieker. 50 Klassische Norddeutsche Gerichte mit Geschichte (“Pot Watcher: 50 Classic North German Dishes with History”).

[83] Sravya (2022). Diwali 2022: 20 Special Deepavali Food Recipes Ideas. https://stylesatlife.com/articles/list-of-diwali-food-recipes/.

[84] Ito H. (2022). Osechi Ryori: Rich in Flavour, Rich in History. https://rafu.com/2022/01/osechi-ryori-rich-in-flavor-rich-in-history/.

[85] Yuan K. (2025). Baked Niangao Sticky Rice Cake. https://blog.themalamarket.com/baked-nian-gao-sticky-rice-cake/.

[86] Kim M. (2024). What Koreans Eat on Seollal Lunar New Year. https://creatrip.com/en/blog/4639.

[87] Guzman C.D. (2023). How Different Countries in Asia Celebrate the Mid-Autumn Festival. https://time.com/6212026/countries-celebrate-mid-autumn-festival/.

[88] Swanick D. (2016). Traditional Wedding Food Enjoyed Around the World. https://danielswanick.com/wedding-food-infographic/.

[89] Rhodes J. Food During Times of Grief. Smithsonian Magazine 2012. https://www.smithsonianmag.com/arts-culture/food-during-times-of-grief-105589529/.

[90] O’Brien S. (2020). A Guide to the World’s Most Comforting Foods of Grief. https://www.atlasobscura.com/articles/funeral-foods-around-the-world.

[91] Ruggeri A. (2017). Kyrgyzstan Bread That Feeds the Dead. https://www.bbc.co.uk/travel/article/20171025-kyrgyzstans-bread-that-feeds-the-dead.

[92] Willcox B.J., Willcox D.C., Todoriki H., Fujiyoshi A., Yano K., He Q., Curb J.D., Suzuki M. (2007). Caloric restriction, the traditional Okinawan diet, and healthy aging: The diet of the world’s longest-lived people and its potential impact on morbidity and life span. Ann. N. Y. Acad. Sci..

[93] Hedenus F., Wirsenius S., Johansson D.J.A. (2014). The importance of reduced meat and dairy consumption for meeting stringent climate change targets. Clim. Change.

[94] Kristbergsson K., Oliveira J. (2016). Traditional Foods: General Comments and Aspects (History, Preparation, Processing).

[95] Alqurashi R.M., Abdall S.M., Ammar A.B., Shatwan I.M., Alsayegh A.A., Alnasser A.N., Alfadhliah J.T., Alnoubi A.A., Fallata G.A., Alhumaidan O.A. (2025). The most popular local and traditional food dishes in different regions of the Kingdom of Saudi Arabia and their cultural significance. Front. Nutr..

[96] Holt V.M. (1885). Why Not Eat Insects?.

[97] Narzary Y., Brahma J., Brahma C., Das S. (2016). A study on indigenous fermented foods and beverages of Kokrajhar, Assam, India. J. Ethn. Foods.

[98] Capiola A., Raudenbush B. (2012). The effects of food neophobia and food neophilia on diet and metabolic processing. Food Nutr. Sci..

[99] Reposo A., Alasqah I., Alfheeaid H.A., Alsharari Z.D., Alturki H.A., Raheem D. (2022). Jellyfish as food. Foods.

[100] Bonaccorsi G., Garamella G., Cavallo G., Lorini C. (2020). A systematic review of risk assessment associoated with jellyfish consumption as a potential food. Foods.

[101] Hsieh Y.P., Leong F.M., Rudloe J. (2001). Jellyfish as food. Hydrobiologia.

[102] Niu J., Ockendon-Powell N.F., Alonge T.A., Papadaki A. (2025). Definition of the African diet. Front. Nutr..

[103] Choi A.S. (2014). What Americans can learn from other food cultures. TED Talk.

